# Sperm Numbers as a Paternity Guard in a Wild Bird

**DOI:** 10.3390/cells11020231

**Published:** 2022-01-11

**Authors:** Melissah Rowe, Annabel van Oort, Lyanne Brouwer, Jan T. Lifjeld, Michael S. Webster, Joseph F. Welklin, Daniel T. Baldassarre

**Affiliations:** 1 Department of Animal Ecology, Netherlands Institute of Ecology (NIOO-KNAW), 6700 AB Wageningen, The Netherlands; A.vanOort@nioo.knaw.nl; 2 College of Science & Engineering, James Cook University, Townsville, QLD 4811, Australia; lyanne@myscience.eu; 3 Division of Ecology and Evolution, Research School of Biology, The Australian National University, Canberra, ACT 2601, Australia; 4 Sex and Evolution Research Group, Natural History Museum, University of Oslo, 0318 Oslo, Norway; j.t.lifjeld@nhm.uio.no; 5 Cornell Lab of Ornithology, and Department of Neurobiology and Behavior, Cornell University, Ithaca, NY 14850, USA; msw244@cornell.edu (M.S.W.); jwelklin@gmail.com (J.F.W.); 6 Department of Biology, University of Nevada Reno, Reno, NV 89557, USA; 7 Department of Biological Sciences, SUNY Oswego, Oswego, NY 13126, USA; daniel.baldassarre@oswego.edu

**Keywords:** fairy-wren, post-copulatory sexual selection, sperm competition, sperm morphology, *Malurus*

## Abstract

Sperm competition is thought to impose strong selection on males to produce competitive ejaculates to outcompete rival males under competitive mating conditions. Our understanding of how different sperm traits influence fertilization success, however, remains limited, especially in wild populations. Recent literature highlights the importance of incorporating multiple ejaculate traits and pre-copulatory sexually selected traits in analyses aimed at understanding how selection acts on sperm traits. However, variation in a male’s ability to gain fertilization success may also depend upon a range of social and ecological factors that determine the opportunity for mating events both within and outside of the social pair-bond. Here, we test for an effect of sperm quantity and sperm size on male reproductive success in the red-back fairy-wren (*Malurus melanocephalus*) while simultaneously accounting for pre-copulatory sexual selection and potential socio-ecological correlates of male mating success. We found that sperm number (i.e., cloacal protuberance volume), but not sperm morphology, was associated with reproductive success in male red-backed fairy-wrens. Most notably, males with large numbers of sperm available for copulation achieved greater within-pair paternity success. Our results suggest that males use large sperm numbers as a defensive strategy to guard within-pair paternity success in a system where there is a high risk of sperm competition and female control of copulation. Finally, our work highlights the importance of accounting for socio-ecological factors that may influence male mating opportunities when examining the role of sperm traits in determining male reproductive success.

## 1. Introduction

In 1970, Geoff Parker [1] fundamentally shifted our understanding of sexual selection. Prior to Parker’s [1] contribution, sexual selection was typically viewed as pre-copulatory competition between males for access to mating opportunities (i.e., male–male competition) and selection of mates by ‘choosy’ females (i.e., female choice). Since, it has become clear that females frequently mate with more than one male during a single reproductive event, and thus selection extends beyond mating as post-copulatory sexual selection in the form of sperm competition (i.e., the competition between sperm/ejaculates from different males for fertilization of a given set of ova [1,2]) and cryptic female choice (i.e., female-regulated fertilization bias towards the sperm/ejaculates of specific males [3,4]).

Sperm competition is a taxonomically widespread phenomenon [5,6] and has significant consequences for male reproductive trait evolution. Indeed, comparative studies across a broad range of animal taxa show that species experiencing high levels of sperm competition (i.e., where multiple mating is common) have larger testes [7,8,9,10,11,12], a greater proportion of sperm-producing tissue in the testes [13,14], and faster and more efficient rates of sperm production [15,16], compared to species in which sperm competition is low. Further, when levels of sperm competition are high, males have longer [12] and faster swimming sperm [17,18], as well as greater proportions of viable and morphologically normal sperm in ejaculates [14,19,20,21] and higher levels of sperm adenosine triphosphate (ATP) [22], see [23]. Finally, sperm length evolves more rapidly when the strength of post-copulatory sexual selection is high [24]. However, while comparative studies provide clear evidence of post-copulatory sexual selection acting on sperm traits, our understanding of how different sperm traits influence male fertilization success remains limited. For example, studies investigating the relationship between the three most commonly assessed sperm traits—sperm morphology, velocity, and viability—and fertilization success report both positive and negative associations, as well as no association between sperm quality and fertilization success (reviewed in [25]).

Birds have been at the center of the study of sperm competition for more than three decades [26,27,28,29,30], likely due to long-standing knowledge of extra-pair mating (i.e., mating outside the social pair bond) and consequent extra-pair paternity (EPP) [31]. Studies of domestic populations have provided key insights into the mechanisms of avian sperm competition [32,33,34,35], as well as the adaptive value of sperm traits. Under competitive conditions, fertilization success is higher for males with relatively greater numbers of sperm [36], longer sperm [37], and higher sperm mobility [38,39,40]. Similarly, artificial insemination studies have shown that sperm motile performance influences male paternity success in mallards (*Anas platyrhynchos*) [41], while both sperm number and mobility shape fertilization success in red junglefowl (*Gallus gallus*) [42]. More recently, a study of red junglefowl held in freely mating groups showed that the male remating rate, mating order and timing of mating (i.e., last male advantage), and male age, as well as female resistance, shape a male’s probability of fertilization success, but that the importance and impact of these factors vary over time [30].

In contrast, studies of wild, free-living avian species have lagged behind those of domestic and captive populations, and have provided mixed results, with some studies finding a link between sperm traits and fertilization success [43,44] and other studies finding none [45,46,47,48]. Multiple explanations could underlie these mixed results. First, the opportunity for selection may be greater in some species than others [47], and indeed, in the two species in which a relationship between sperm traits and paternity success has been reported, extra-pair mating is common and subsequent rates of EPP are high ([43,44], reviewed in [47]). Second, a recent simulation study demonstrated that the use of parentage data to infer selection on sperm traits can lead to highly biased results under many common statistical approaches, especially when traits linked to pre-copulatory sexual selection are not taken into account [49]. Additionally, we suggest that it can be informative to take into account extrinsic ecological factors that may constrain the ability of individuals to engage in extra-pair mating. For example, breeding density is thought to influence encounter rate between individuals, with higher encounter rates expected to facilitate extra-pair mating opportunities [27,50]. Similarly, social factors, such as the relatedness of social pairs or group size/composition, may favor infidelity or influence mating decisions [51,52,53]. Thus, a range of socio-ecological factors may influence male mating opportunities both within and outside the social bond, and thus it is useful to take into consideration such factors when trying to understand how selection acts on sperm traits.

Among the passerine birds, the Maluridae (fairy-wrens, emu-wrens, and grasswrens), and the fairy-wrens (genus *Malurus*) in particular, are emerging as a model system for the study of sexual selection and sperm competition. These species exhibit a broad range of rates of EPP [54,55,56], and a range of apparent adaptations to high levels of sperm competition, including relatively large testes, more efficient sperm producing tissue in the testes, and high rates of sperm production, as well as large numbers of high- quality sperm in sperm reserves [14,55,57]. Female fairy-wrens appear to exhibit strong female choice of extra-pair mating partners based on male plumage traits [58,59,60,61]. In addition, the Maluridae is probably the best studied avian family with respect to socio-ecological drivers of variation in EPP. A recent study examining variation in EPP in 20 populations of nine species over 89 years, showed that females had higher rates of EPP in the presence of more helpers, when they were surrounded by a greater number of neighbors, or when they were paired incestuously [56].

In this study, we tested for an effect of sperm quantity and sperm morphology on male reproductive success in the cooperatively breeding red-backed fairy-wren (*M. melanocephalus*), while simultaneously accounting for the potential effects of pre-copulatory sexually selected traits and socio-ecological factors that may influence individual mating opportunities. The red-backed fairy-wren is ideal for this work for a number of reasons. First, red-backed fairy-wrens likely experience strong postcopulatory sexual selection as rates of EPP are high: 56% of all offspring are sired by extra-pair males and 67% of all broods contain at least one extra-pair young [62]. Second, male plumage coloration is a target of pre-copulatory sexual selection. Breeding males typically display one of two distinct nuptial plumage phenotypes: red/black vs. brown. Notably, the more colorful red/black males are preferred by females, engage in more extra-territorial forays, and gain a greater share of paternity success, while brown breeders spend more time both physically and acoustically guarding their social female [59,60,62,63]. Third, multiple socio-ecological factors are known to influence among-individual variation in EPP in this species; female red-backed fairy-wrens show greater levels of EPP when paired incestuously [56,64] and when there are helpers present in the social group, presumably relaxing the constraints for females to engage in EPP [56]. Finally, by studying a species that lays few eggs (i.e., mean clutch size of 3), we minimize the potential for temporal effects of sperm traits on male fertilization success. Given putative low copulation rates in fairy-wrens, Rowe and Pruett-Jones [14] proposed that male fairy-wrens may maximize paternity success via transfer of large numbers of sperm in one or a few ejaculates. Therefore, we predicted that sperm numbers would be under selection in the red-backed fairy-wren. Based on prior findings in the superb fairy-wren (*M. cyaneus*) [44], we also predicted that paternity success would be associated with the sperm flagellum:head ratio, with males whose sperm have a relatively longer flagellum and short head gaining more within-pair paternity (WPP), and those whose sperm have a relatively shorter flagellum and longer head gaining EPP. Similarly, because longer sperm have been shown to fertilize more eggs in captive zebra finches (*Taeniopygia guttata*) [37], we tested for an association between total sperm length and paternity success in our population. Finally, given evidence of stabilizing selection on sperm length in passerine birds [65,66,67], we asked whether male reproductive success was associated with either an ‘optimal’ sperm length or a reduction in variation in total sperm length.

## 2. Materials and Methods

### 2.1. Study Species, Population Monitoring, and Sample Collection

We studied a population of red-backed fairy-wrens at Lake Samsonvale (27°16′ S, 152°51′ E), 30 km northwest of Brisbane, Queensland, Australia. We extensively monitored the population for three breeding seasons (from September to January) during 2010–2014. During one breeding season (September 2011–January 2012), a manipulative experiment was carried out that altered patterns of EPP within the population [68]; thus, only samples and data collected for two breeding seasons (hereafter referred to as 2011 and 2013) were used in the current study. In this population, groups consist of a dominant breeding pair, which may or may not be joined by one or more male helpers that assist with feeding nestlings and fledglings. Groups with helpers are relatively infrequent, however, with just 12.7% (9/71) and 11.0% (14/127) of groups including helpers in 2011 and 2013, respectively. Furthermore, when groups do include helpers, the number of helpers is typically low (mean ± SD: 1.33 ± 0.5 (range: 1–2) and 1.36 ± 0.75 (range: 1–3) in 2011 and 2013, respectively).

During our study, we captured, color-banded, and took blood samples from adults, and found all nests in order to band and collect blood samples from all nestlings at 6 days of age. Adult birds were captured using mist nets set on their home territories, and upon capture we collected standard morphological measurements (e.g., body mass). For males, we also measured the length, width, and depth of the cloacal protuberance, and the volume of the cloacal protuberance was calculated as volume = π × D/2 × W/2 × L [69]. In addition, we scored plumage coloration of males following [70], classifying males as red/black (≥67% plumage red/black scored on six body regions), intermediate (between 33% and 66% plumage red/black), or brown (≤32% plumage red/black). As previously documented [62], we found that plumage coloration was strongly bimodal and the majority of males (98%) were classified as either red/black or brown. Male plumage coloration is associated with age in this species; 24% of 1-year-old males exhibit red/black plumage, whereas 96% of 2-year-old males and 100% of 3-year-old males exhibit red/black plumage [71]. However, we were not able to unambiguously assign age to many of the individuals used in the current study. This was because the majority of individuals were first captured as adults and were thus classified as after second year, though a small number of individuals (*n* = 23) were aged based on the degree of skull ossification or from nestling banding records (2013 only). Given this, we do not consider male or female age in our subsequent analyses. For males, we also collected a sperm sample via cloacal massage [72,73]. Immediately after collection, sperm were diluted in 20 μl of phosphate buffered saline (PBS) and then this diluted sample was fixed in 5% formaldehyde for later morphological analysis.

### 2.2. Sperm Morphology and Sperm Numbers

We collected a total of 141 sperm samples from 130 individual males during the two focal breeding seasons. To measure sperm morphology, a 10 μl aliquot of the fixed sperm sample was spread evenly across a glass slide, allowed to air dry overnight, and then gently rinsed with distilled water and again allowed to air dry and stored for later analysis. We then imaged sperm cells at 200× magnification using bright field microscopy (acA1300–200uc Basler camera connected to a Nikon Eclipse E200 light microscope) and digital image capture (pylon viewer, Basler). We aimed to measure length of the sperm head, midpiece, and tail (i.e., exposed flagellum) of 20 morphologically normal and intact sperm cells per male using SpermSizer v1.6 [74], though for some males this was not possible (number of measured cells, mean ± SD: 21.3 ± 6.2, median: 21, range: 1–50, number of males with fewer than 10 measured cells = 2). For each cell, these measurements were used to obtain values of total sperm length (sum of head, midpiece, and tail), flagellum length (sum of midpiece and tail), and the ratio of flagellum to head length. All measurements were taken by the same observer (AvO), who demonstrated high repeatability (R) of sperm measurements, when measuring 70 sperm cells twice blind to the previous measurement (repeatability: head length = 0.93, midpiece length, 0.94, tail length = 0.92, and total sperm length = 0.99; [75]).

We assessed intra-specific variation in sperm morphology by calculating both among-male and within-male coefficient of variation [76] in sperm length. Specifically, we calculated the among-male coefficient of variation (CVam) of total sperm length using data from a single sperm sample from each of the 130 males using the formula CV = (SD/X) × 100, where SD is the standard deviation of mean total sperm length and X is mean total sperm length. Next, we calculated the within-male coefficient of variation (CVwm) of total sperm length. In this instance, we excluded any males with fewer than 10 measured sperm cells. Additionally, given that the CV will be underestimated for small sample sizes, we corrected the CVwm according to the formula: Adjusted CV = ((SD/X) × 100) × (1 + (1/4 N)), where N is the number of cells measured per male [76].

We used cloacal protuberance volume as a proxy for sperm numbers; cloacal protuberance volume has been shown to correlate with both testis size and sperm numbers in storage in the seminal glomera both within and across passerine species generally [77,78,79], as well as within and across species of fairy-wren [14,69]. Thus, although it is unknown whether cloacal protuberance volume reflects the number of sperm in natural ejaculates, it does reflect the number of sperm in sperm stores, and thus sperm numbers available for copulation(s). Given that cloacal protuberance volume may vary across the breeding season, we tested for an association between measurement date and cloacal protuberance volume; this showed no association between measurement date and the volume of the cloacal protuberance (β = 0.009 ± 0.007 (SE), t = 1.16, *p* = 0.25).

### 2.3. Genetic Paternity Analysis

We extracted DNA from blood samples using the Omega Bio-Tek EZ-96 Total DNA/RNA Isolation Kit^®^. We then genotyped all individuals at seven highly polymorphic microsatellite loci developed from different bird species (Table S1). PCR products were separated on an ABI Prism 3730^®^ automated sequencer, and alleles were scored using the program GeneMapper (Applied Biosystems) and verified by eye. We assigned paternity using the program CERVUS 3.0 [80], which determines which male in the population has the highest likelihood of siring a given offspring. CERVUS calculates a log likelihood score (LOD) for each male accounting for offspring genotype, maternal genotype, and genotype scoring errors (e.g., from null alleles). For each assignment, we used a “total evidence” [81] approach to check the CERVUS assignment. Using this additional evidence likely improved the reliability of several assignments but was unlikely to affect our results because we accepted the CERVUS-assigned male in most cases. When combined, the microsatellite loci were highly polymorphic and informative for paternity analysis (mean number of alleles per locus = 11.7, mean expected heterozygosity = 0.69, Table S1). Allele frequencies did not deviate from Hardy–Weinberg expectations, but two loci (*Mcy2* and *Smm7*) had an estimated null allele frequency greater than 0.05, which was accounted for in subsequent paternity assignments. The average probability of excluding a randomly chosen male as the sire was high, with a combined exclusion probability of 0.998 See Supplementary Materials for the full details of the genetic paternity analysis.

### 2.4. Socio-Ecological Factors

In order to account for the potential role of socio-ecological factors in shaping variation in paternity success among males, we determined: (1) if there were helpers present at the nest during a breeding event (classified as Yes/No), (2) the number of immediate neighboring territories (as a proxy for the number of neighboring females), and (3) whether the social-pair were close relatives (i.e., paired incestuously) or not. The presence of helpers was determined through detailed field observations undertaken throughout the breeding season. The number of immediate neighboring territories was calculated for each focal territory through the use of territory maps. Territory maps were created by combining GPS coordinates of territory centers, edges, and nest locations with detailed field observations of territory boundaries and group composition and interactions.

To determine whether a pair was incestuous or not, we estimated relatedness (*r*) by calculating the pairwise *r* according to Wang [82], which has been shown to perform well for highly polymorphic loci, using the program COANCESTRY [83]. This metric scales from −1 to 1, with first-order relatives such as parents and offspring having an *r* value of 0.5. We first assessed the quality of our molecular data by calculating relatedness scores for all known first-order relatives (i.e., mother–offspring combinations). We found that known first-order relatives exhibited a mean pairwise *r* of 0.45 ± 0.15 (range −0.08 to 0.91, *n* = 420 mother–offspring combinations, Figure S1). Based on the graphical distribution of these pairwise relatedness values, and given that our pairwise *r* measures were somewhat underestimating relatedness, we considered a pair as incestuous when its pairwise *r* was ≥0.375 (*n* = 8 pairs). We also consider an alternative incestuous pair cut-off criterion following Brouwer et al. [56] as *r* ≥ mean ± 1.5 SD of known first-order relatives. Under this criterion, we considered a pair as incestuous when its pairwise *r* was ≥ 0.243 (*n* = 41 pairs). We then calculated pairwise *r* for all social pairs recorded in our population across the entire study period (2010–2013). This showed that the relatedness of social pairs was generally low (mean ± SD = 0.0296 ± 0.2, *n* = 221 pairs, Figure S2). Across the population as a whole, the proportion of incestuous pairs in the population tended to be higher in 2011 than 2013 (*r* ≥ 0.375 = 0.048 vs. 0.039, one-sided binomial test *p* = 0.42; *r* ≥ 0.243 = 0.197 vs. 0.109, one-sided binomial test *p* = 0.005). We focus on the analyses using the cut-off of *r* ≥ 0.375 in the main text.

### 2.5. Statistical Analyses

We assessed the relationship between male paternity success and multiple sperm traits, including total sperm length as both a linear and quadratic term (the latter to test for the possibility of stabilizing selection on sperm length), the standard deviation of total sperm length (to test for the possibility that males with less-variable sperm length gain greater paternity success), sperm flagellum:head length ratio, and cloacal protuberance volume (as a proxy for sperm numbers). For all sperm traits, we used average values from each unique sperm sample, and only males with at least 10 measured sperm cells (mean± SD: 17.6 ± 6.7, median: 16) were included in analysis of paternity. All variables were scaled to z-scores before including them in the model to facilitate model convergence. We considered paternity success as three separate measures: (1) total paternity success, (2) within-pair paternity (WPP) success, and (3) extra-pair paternity (EPP) success.

For total paternity success, we used a generalized linear model with negative binomial error distribution and modelled total paternity success as the total number of offspring sired (i.e., the number of offspring sired in the social nest plus the number of offspring sired in other nests). In addition to sperm morphological traits and cloacal protuberance volume, we included male breeding plumage (red/black vs. brown), presence of helpers (yes/no), number of neighbors, and incestuous pairing (yes/no) as covariates in our model. We accounted for potential variation between the two focal years by including year as a fixed factor. Because red/black breeding males exhibit larger cloacal protuberance volumes than brown breeding males [72], we also tried fitting a model that included an interaction between cloacal protuberance volume and male breeding plumage in order to determine whether an effect of sperm numbers depended upon male breeding phenotype. Inclusion of the interaction term increased the model AICc value (ΔAICc = 3.07), and a likelihood ratio test supported dropping the interaction term from the model (LR = 0.02, *p* = 0.89). Thus, we did not include the interaction term in our final global model. For this analysis, we excluded males that lacked paternity data at their social nest, regardless of whether or not they gained EPP in other nests, because in such instances a zero value for WPP does not accurately reflect a male’s success or lack thereof but may instead be driven by other factors such as predation events. The resulting dataset included total paternity success for 67 males. One male had data in both study years, though in both years this male’s paternity success was zero. We therefore chose one record at random for our analysis. Next, in our dataset, one male exhibited a very large cloacal protuberance volume. We therefore repeated our analyses with this individual excluded to ensure our results were robust. Finally, we also ran a model of total paternity success, in which we modelled total paternity success as a binomial response (sum of all offspring sired across all broods vs. sum of offspring not sired by the focal male across all broods) in a generalized linear model with logit link function. We used this approach to examine a male’s success when in competition with other males.

Next, we examined WPP success using a generalized linear mixed model with logit link function and bobyqa optimization [84], with WPP success fitted as a binomial response (number of WP young vs. number of EP young in a brood). To account for some social pairs in our dataset having multiple broods in a breeding season, we included pair identity (male + female identity) as a random effect to account for non-independence of the data. In addition to sperm morphological traits and cloacal protuberance volume, we included male breeding plumage (red/black vs. brown), presence of helpers (yes/no), number of neighbors, and incestuous pairing (yes/no) as covariates in our model. We accounted for potential variation among the year by including year as a fixed factor. As before, we also tried fitting a model that included an interaction between cloacal protuberance volume and male breeding plumage to determine whether an effect of sperm numbers depended upon male breeding phenotype. Again, inclusion of the interaction term increased the model AICc value (ΔAICc = 1.73), and a likelihood ratio test supported dropping the interaction term from the model (Chi sq = 1.19, *p* = 0.28). Thus, we did not include the interaction term in our final global model. Additionally, we repeated our analyses excluding the one male with the extreme value for cloacal protuberance volume to check the robustness of our results. For these analyses, we only included males that successfully raised a brood (i.e., day 6 nestlings) with their social female. The resulting dataset included 76 broods from 67 individual males.

For EPP, we again used a generalized linear model with logit link function. We modelled EPP as a binomial response (number of nestlings sired by a male outside of the social bond summed across all nests vs. number of offspring not sired by a male in these same nests). In addition to sperm morphological traits and cloacal protuberance volume, we included presence of helpers (yes/no) and number of neighbors as covariates in our model. We did not include incestuous pairing in this main model as no pairs were classified as incestuously paired at the cutoff of *r* ≥ 0.375 We also accounted for potential variation between focal years by including year as a fixed factor. For this analysis, we only included males that sired one or more EP offspring following [49], because males that fail to sire at least one EP young cannot be assumed to be in competition for fertilization. This resulted in a dataset of 31 males. However, just 1/31 of these males exhibited brown breeding plumage, indicating an extremely strong bias towards males in red/black breeding plumage siring EP young. We therefore limited our data only to males in red/black breeding plumage (*n* = 30).

For all three models we used a model selection approach, with all possible combinations of predictors, and ranked models by Akaike’s Information Criterion corrected for sample size (AICc) [85], with lower AICc values being considered as better supported by the data. In addition, we report normalized Akaike weights to assess the relative support for competing models [86]. Modelling assumptions of all global models and top models following model selection were examined using the package DHARMa [87], which showed that the models fit the data well. Marginal and conditional *R*^2^ [88] were calculated to determine the relative importance of the predictors for explaining variation in paternity success. Collinearity was examined using variance inflation factor analyses in the package CAR [89], which indicated very low collinearity among predictors (VIF < 1.72, [90]). Results of the global models were highly congruent with findings using the model selection approach (Tables S2–S4). All statistical analyses were performed using R version 4.1.0 [91] using R studio [92] and packages lme4 [93] and MuMIn [94]. 

## 3. Results

### 3.1. Genetic Parentage

The 2011 dataset contained 89 genotyped offspring. Of these, we were able to assign paternity to 77 (86.5%) and accepted the CERVUS-assigned male 98.7% of the time. Twelve additional offspring were identified as extra-pair offspring, but we could not assign a sire. In 2011, 64.1% (57/89) of the young were extra-pair offspring, and 73.2% of broods (30/41) contained at least one extra-pair young. The 2013 dataset consisted of 186 genotyped offspring. Of these, we were able to assign paternity to 181 (97.3%) and accepted the CERVUS-assigned male 77.9% of the time. Five additional offspring were identified as extra-pair offspring, but we could not assign a sire. In 2013, 47.3% (88/186) of the young were extra-pair offspring, and 57.9% of broods (44/76) contained at least one extra-pair young.

### 3.2. Sperm Morphology and Cloacal Protuberance Volume in the Red-Backed Fairy-Wren

**Figure 1:**
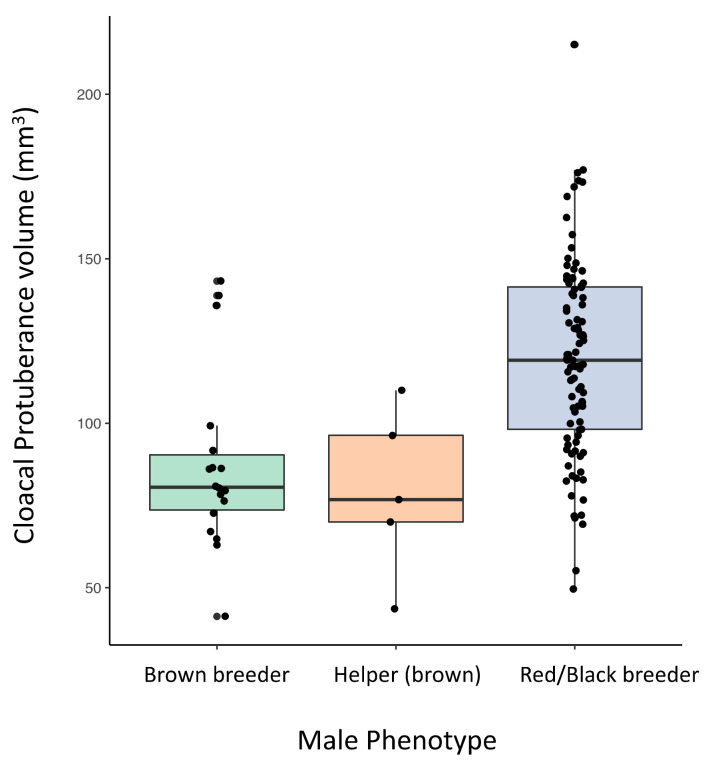
Cloacal protuberance volume in the three male phenotypes in the red-backed fairy-wren: red/black breeders (*n* = 96), brown breeders (*n* = 18), and helpers (*n* = 5). Breeding males in red/black breeding plumage had a significantly larger cloacal protuberance volume compared to both breeding males in brown plumage and helper males (also in brown plumage). See main text for full statistical details.

Across all 130 males, total sperm length averaged 89.34 μm ± 1.94 μm (mean ± SD) and the among-male coefficient of variation in total sperm length (CVam) was 2.17, while the within-male coefficient of variation in total sperm length (CVwm) was 2.08. Total sperm length did not differ among the male phenotypes (F_2,121_ = 2.01, *p* = 0.14, Table S5). Sperm midpiece length and flagellum length did not differ among the male phenotypes (midpiece: F_2,121_ = 1.25, *p* = 0.29; flagellum: F_2,121_ = 0.40, *p* = 0.68), whereas sperm head length was significantly shorter in helper males compared to both red/black (*p* = 0.006) and brown (*p* = 0.02) breeders (Table S5). This difference in head length also translated into slight differences in F:H ratio among the phenotypes (F_2,121_ = 3.15, *p* = 0.047; Table S5). Finally, cloacal protuberance volume differed significantly across the male phenotypes (F_2,116_ = 12.96, *p* < 0.001, Figure 1); red/black breeders had a larger cloacal protuberance volume compared to both brown breeders (*p* < 0.0001) and helper males (*p* = 0.008), but it did not differ between brown breeders and helper males (*p* = 0.85).

### 3.3. Sperm Traits and Paternity Success

Over the two focal years of our study, we observed substantial variation in total paternity success, with males siring on average 2.2 ± 2.2 (SD) offspring (range 0–8). Total paternity success (i.e., sum of WPP and EPP) was strongly associated with the three socio-ecological factors included in our models: presence of helpers, number of neighbors, and whether or not pairs were incestuous (Table 1, see Table S6 for full model output). Males had higher paternity success when helpers were present in the social group and when the number of neighbors was higher, but lower paternity success when paired incestuously (model 1, Table 1). We also observed a significant effect of year, with total paternity success being higher in 2013 compared to 2011 (Table 1). Similarly, male nuptial plumage coloration was associated with total paternity success; plumage coloration was included in the top 24 models (Table S6). Specifically, males in red/black breeding plumage gained greater paternity success relative to males in brown plumage. In contrast, we found no support for an association between sperm traits and total paternity success in our main analyses, as adding these variables to the top model increased AICc values (e.g., models 2 and 3 vs. 1, Table 1). However, when we excluded the single male with an extreme value of cloacal protuberance volume, we found some support for an association between sperm numbers and total paternity success (e.g., top model, Table S7). In our main analysis, our analyses returned similar results when we set the relatedness cut-off to *r* ≥ 0.243 (Table S8). Similarly, when we modelled total paternity success as a male’s relative success when in competition with other males (i.e., sum of all offspring sired across all broods vs. sum of offspring not sired by the focal male across all broods), the results were qualitatively similar to the main analyses (Table S9).

**Figure 2:**
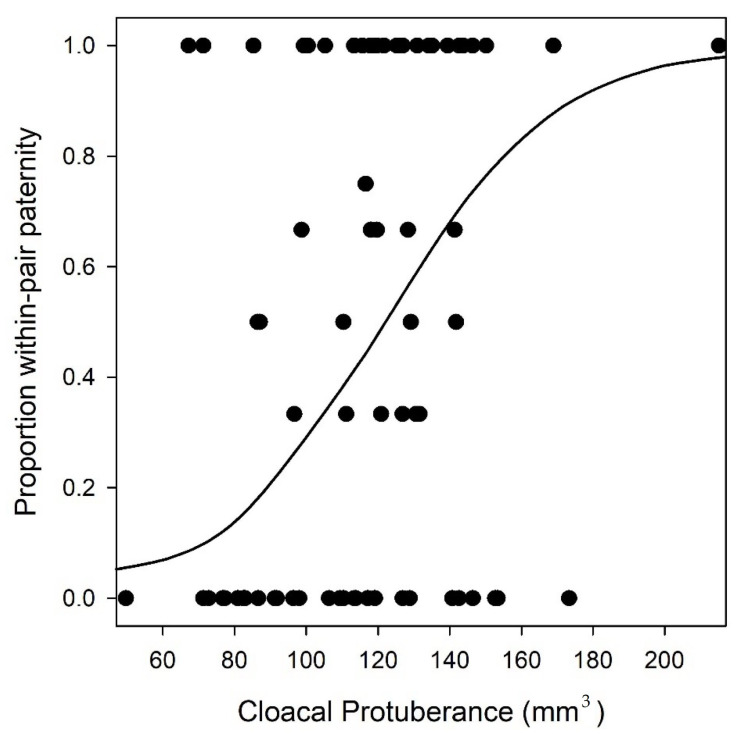
The relationship between a male’s cloacal protuberance volume and the proportion of within-pair paternity in a brood in the red-backed fairy-wren (*n* = 76). The trendline shows the predictions according to model 1 (Table 1).

Next, WPP success was strongly associated with the volume of the cloacal protuberance; cloacal protuberance volume was consistently included in the top 184 models (Figure 2, Table 2, see Table S10 for full model output). In contrast to our predictions, a lower F:H ratio was associated with higher WPP success, indicating that males with relatively shorter sperm flagellum and longer sperm head had greater WPP success. However, it should be noted that support for this association was weak and confidence intervals overlapped zero (ΔAICc = 0.4, Table 2, model 1 vs. 2). We found little support for the idea that total sperm length or within-male variation in total sperm length was associated with WPP success, as adding these variables to the top model increased AICc values (Table 2). Similarly, none of the socio-ecological factors received model support (Table 2). Although a large proportion of the conditional (through random and fixed effects) variance in WPP was accounted for in the models (85%), the amount of marginal variance in WPP that could be attributed to cloacal protuberance and F:H ratio was much lower (22%), with most of this explained by cloacal protuberance (17%). Analysis of the reduced dataset (i.e., excluding the single male with an extreme value of cloacal protuberance volume) returned qualitatively similar results (Table S11). When the analysis was run with the alternative cut-off for an incestuous pairing (i.e., *r* ≥ 0.243), the results were similar, though in this case, whether or not a male was paired incestuously also influenced WPP; males paired incestuously had lower WPP paternity success (Table S12).

Extra-pair paternity success was strongly associated with male breeding plumage coloration. That is, 97% (30/31) of the males with EPP in our dataset exhibited red/black breeding plumage. We therefore examined whether sperm traits influenced variation in EPP success among red/black breeding males only. We found that EPP varied between the study years, being higher in 2011 compared to 2013 (Table 3, see Table S13 for full model output). In contrast to our predictions, there was no evidence that any sperm traits were associated with EPP success in red/black breeding males, as adding these variables to the model increased AICc values (e.g., models 2 and 4, vs. 1, Table 3). None of the socio-ecological variables explained any of the variation in EPP success among red/black breeding males when r ≥ 0.375 (Table 3). However, when we set the relatedness cut-off to *r* ≥ 0.243, we found some support for socio-ecological variables to explain variation in EPP; EPP success was greater when males were paired incestuously, and lower when the male’s social group included helpers (Table S14).

## 4. Discussion

Understanding how selection imposed by sperm competition shapes sperm traits and the role of sperm in fertilization success has been a major focus of both theoretical (reviewed by [95]) and empirical (reviewed by [25]) studies. However, while birds have been the focus of sperm competition studies for decades [29], our understanding of how sperm traits influence male reproductive success in natural populations remains limited. Here, by exploiting a vast body of knowledge concerning pre-copulatory female mate choice and socio-ecological factors driving mating decisions in the Australian fairy-wrens, we were able to examine the role of sperm traits in determining male paternity success while taking into account factors that may influence a male’s access to mates. We found that sperm numbers, but not sperm morphology, influences reproductive success in wild, free-living, male red-backed fairy-wrens.

Most notably, our analyses revealed that sperm numbers were the major driver of variation in WPP. Specifically, male red-backed fairy-wrens with large numbers of sperm available for copulations were more successful at gaining WPP than males with relatively small sperm reserves. Sperm competition theory predicts that males should increase their investment in sperm numbers when the risk of sperm competition is high [95,96,97,98]. This is especially true in relatively large-bodied, internally fertilizing animals, such as birds, when males compete under a raffle model of sperm competition and when males cannot monopolize females [99,100]. In birds generally, sperm competition is thought to obey the raffle principle with passive sperm loss [30,33,100], while the mating behavior of fairy-wrens suggests female monopolization is generally low. Specifically, a large proportion of females have at least one extra-pair offspring in a brood, suggesting that males are generally unable to prevent female multiple mating. Indeed, in the superb fairy-wren, and presumably other fairy-wrens, females control extra-pair mating opportunities by conducting pre-dawn forays to copulate with males outside of the social group [101]. Moreover, upon return to their home territory, females copulate with their social partner [54]. If sperm competition forms a raffle, the social male may gain or guard WPP through a numerical advantage if large numbers of sperm are transferred during copulation [33,95]. Given passive sperm loss, the social male may also gain a fertilization advantage as the last male to mate [33,95], though in this specific situation the time delay between matings is minimal, and thus, a last male advantage resulting from passive sperm loss is also expected to be minimal. Alternatively, though not mutually exclusive, the post-foray within-pair copulation may suggest that the social male can detect the occurrence of an extra-pair mating and ‘knows’ he occupies a disfavored mating role. Under this scenario, sperm competition follows a loaded raffle and social males are predicted to increase sperm allocation to ejaculates to gain paternity [95,96,102].

Selection for large numbers of sperm appears to be a common evolutionary response to sperm competition [12]. However, large numbers of sperm may be allocated to either a few large ejaculates or to many small ejaculates [6,103,104]. Theoretical models of sperm expenditure predict that sperm investment into individual ejaculates can depend on both the level of sperm competition (i.e., risk or intensity of sperm competition) and male mating rate. Specifically, when the probability of double mating by females is high (risk model), males should invest in large numbers of sperm per ejaculate to gain a numerical advantage in sperm competition, whereas sperm numbers per ejaculate are predicted to decrease when females mate with more than two males (intensity model) [98,105,106]. At the same time, when male mating rate is high, large numbers of sperm allow a male to produce a greater number of smaller ejaculates to engage in frequent copulations and avoid sperm depletion [104,107]. Available evidence suggests that fairy-wrens face a high risk of sperm competition and likely allocate large numbers of sperm to a few ejaculates. First, mating rate is generally thought to be low in fairy-wrens as copulations are rarely observed [54]. Second, in our study population, the average number of sires per clutch was 1.4 (2011: mean 1.4 ± 0.5 sd, range 1–2; 2013: mean 1.4 ± 0.6 sd, range 1–3, Baldassarre, *unpublished data*), although these estimates are based on genetic parentage and thus may underestimate the actual number of female mating partners. Intriguingly, previous studies of the red-backed fairy-wren have shown that males in red/black vs. brown plumage are not cuckolded at different rates [62]. This has been attributed to, at least in part, the fact that brown breeders spend more time physically and acoustically mate guarding their social female [59,63]. The current study builds on this work to shown that males with larger cloacal protuberances (and hence larger sperm reserves) may also physiologically guard their social females via the transfer of large numbers of sperm during copulation. Indeed, although we currently lack information on female-driven post-copulatory processes in our system, we suggest that large numbers of sperm in an ejaculate are a general male defensive strategy to guard WPP under high risk of sperm competition and female control of copulations.

In contrast to our findings for WPP, we found that both total paternity and EPP success was strongly influenced by male plumage coloration, with males exhibiting red/black breeding plumage siring more offspring that males breeding in brown plumage. For EPP, this was evidenced by only a single brown breeding male in our dataset gaining EPP in the two years of our study. These findings are highly consistent with previous studies demonstrating that plumage color (red/black vs. brown) is a target of pre-copulatory female mate choice and subject to sexual selection in the red-backed fairy-wren [59,60,62]. At the same time, red/black breeding males have a larger cloacal protuberance and thus greater numbers of sperm available for copulation (this study, [71]). Moreover, when we ran our analysis excluding the single male with an extreme value of cloacal protuberance volume, we found that sperm numbers were positively associated with total paternity success. Although female-driven post-copulatory processes may also play a role in shaping red/black male reproductive success, our results suggest that red/black males may have high reproductive success, at least in part, because they gain extra-pair mating opportunities via pre-copulatory female choice and/or because they gain fertilizations via a numerical advantage from large numbers of sperm in an ejaculate.

Among red/black breeding males, however, variation in EPP success did not appear to be associated with sperm number or any sperm morphological traits. One possibility is that male traits other than those assessed in our study may contribute to variation in EPP success. Previous work in this same population of red-backed fairy-wrens found no effect of natural plumage hue (i.e., redness) on male reproductive success [68]. In the superb and red-winged fairy-wrens, however, the timing of molt into nuptial plumage has been shown to be a predictor of EPP success [58,61,108]. Unfortunately, we lack information on molt date for our study and thus could not include the potential effects of molt initiation date in our analyses. Similarly, earlier studies, while suggesting that molt date may be an important sexually selected trait in the red-backed fairy-wren, lack the necessary data to explicitly test the effects of molt initiation date on male reproductive success [59]. Alternatively, it is possible that only males with a sufficiently large numbers of sperm (i.e., red/black breeding males) available for copulation gain EPP and any further variation in sperm number does not further improve success. Finally, the potential contribution of female-driven post-copulatory processes to paternity outcomes may obscure any associations between ejaculate traits and EPP success.

Although we were unable to link any male traits to variation in EPP success within this class of males (i.e., red/black breeders) in our study, we did observe a clear difference in EPP between the two study years. Specifically, EPP rates were higher in 2011 than in 2013. In fairy-wrens generally, temporal variation in EPP rates has been linked to annual variation in the proportion of incestuous pairs within a population, presumably due to female avoidance of inbreeding via extra-pair copulations [56]. Here, we also found evidence for a link between EPP rates and inbreeding avoidance; the year with higher EPP rates (2011) also had a greater proportion of incestuous pairs in the population as a whole. Moreover, we found that total male reproductive success was strongly influenced by whether or not a male was paired incestuously; males paired with a related female had lower total paternity success compared to males paired with an unrelated female. In the red-backed fairy-wren, there is strong correlational and experimental evidence that females paired to genetically similar males are more likely to produce offspring sired by extra-pair males, and thus females avoid the potential costs of inbreeding through extra-pair copulations [64,109]. Studies of other fairy-wren species further demonstrate that female avoidance of inbreeding is an important factor contributing to variation in EPP among individuals [56,61]. Thus, our findings are consistent with previous studies pointing to the importance of inbreeding avoidance as a driver of individual mating decisions and influencing among individual and temporal variation in EPP in the red-backed fairy-wren.

Total male reproductive success was also strongly associated with multiple socio-ecological factors which may be linked to male mating opportunities. First, males achieved greater paternity success when the number of adjacent neighboring territories was higher, which is consistent with previous studies of fairy-wrens [56] and may arise as a result of increased encounter rates between potential mating partners (*sensu* the density hypothesis [27,50]). Second, males achieved greater paternity success when the male’s social group included helpers. In red-backed fairy-wrens, breeding males with helpers show reduced offspring provisioning rates relative to males without helpers [109,110], suggesting that the presence of helpers may allow males to shift effort away from parental care to other activities, including activities related to the acquisition of extra-pair matings [111]. However, our study contrasts with previous studies of fairy-wrens that have shown no effect of the presence of helpers on male reproductive success [110,111,112], as well as a decrease in paternity success with an increasing number of helpers [113]. This discrepancy may be due to methodological differences between studies, e.g., in this study we considered the presence of helpers as a yes/no response, whereas other studies use the number of helpers (e.g., [113]). Alternatively, the effect of helpers observed in our study might arise via associated effects of territory or female quality, as has been proposed for findings in the superb fairy-wren [111], or effects of other ecological variables correlated with the presence of helpers (e.g., availability of vacant territories) rather than as a direct consequence of helpers per se.

Intriguingly, in our study we found little evidence that sperm morphological traits influence male reproductive success in the red-backed fairy-wren. This contrasts with a study of the superb fairy-wren, which demonstrated that lifetime EPP success was associated with sperm with a shorter flagellum and relatively large head, while WPP success was associated with sperm with a longer flagellum and relatively short head [44]. In the same study, Calhim et al. [44] reported a two-fold difference between the inter- and intra-male variation in total sperm length in the superb fairy-wren. In contrast, we observed little difference between these two values (2.17 vs. 2.08, inter- and intra-male variation, respectively) in the red-backed fairy-wren. Thus, one possible explanation for these contrasting results may be differences between the species in dynamics of sexual selection and sperm competition. Rates of EPP are somewhat higher in the superb fairy-wren compared to the red-backed fairy-wren: broods containing EP young: 92% superb [114] vs. 67% red-backed [62] fairy-wren. Thus, post-copulatory offensive and defensive paternity strategies may be under stronger selection in the superb fairy-wren. Alternatively, in our study, we tested for associations between sperm morphology and reproductive success within a single breeding season, whereas Calhim et al. [44] assessed reproductive success over a male’s lifetime (accounting for variation in lifespan). Finally, differences in sperm measurement approaches may contribute to observed differences between these studies. First, Calhim et al. [44] measured sperm collected from the liquid part of a fecal sample, whereas we collected sperm via cloacal massage. While a study of the zebra finch suggests that sperm collected from feces is reliable for the assessment of sperm morphology [115], a study of house sparrows reported a significant difference in sperm morphology measurements obtained from the two methods [116]. Second, we measured a larger number of sperm cells per male in the current study (mean sperm per male: 17.6 vs. 7; this study vs. [44], respectively). The number of sperm measured per male can influence estimates of total sperm length and the coefficient of variation in total sperm length in passerine birds [66,117], and thus may also impact the accuracy of flagellum:head ratio estimates. Although it is unclear how collection method or sample size might influence measurements of sperm flagellum:head ratio in fairy-wrens, it is nonetheless possible that methodological differences contribute to the differences between studies.

Finally, our findings contribute to our understanding of sperm evolution in the Maluridae. Calhim et al. [44] highlighted that the short sperm of the superb fairy-wren contrasted with studies showing that sperm length is a target of post-copulatory sexual selection [12,118]. Similarly, in comparison to the variation observed across the songbirds (Aves: Passeri: 40–290 μm [119]), we find that red-backed fairy-wren males also have relatively short sperm (89.34 μm) despite high levels of post-copulatory sexual selection. Relatively short sperm is also characteristic of other Australian Maluridae (75.43–90.63 μm, Rowe *unpublished data*, Table S15). More generally, within the songbirds, longer sperm are typically found in the Passerida, while short sperm are characteristic of the Corvoidea [120], and our findings suggest sperm may also be relatively short in the Meliphagoidea. Given the importance of sperm numbers for paternity success suggested by the findings of this study and the documentation of large testes and high rates of sperm production in a range of fairy-wren species [14,55,57], we suggest that the fairy-wrens generally experience strong selection for sperm numbers. In contrast, evidence for either directional or stabilizing selection on total sperm length is currently weak in the fairy-wrens (this study, [44]). Intriguingly, however, contrasting findings concerning the role of the sperm flagellum:head length ratio in shaping male reproductive success (this study, [44]), suggests that selection on some aspects of sperm morphology may be variable across different fairy-wren species. Thus, we suggest that additional studies of sperm traits in this enigmatic group of birds will further contribute to our understanding of the role of sperm traits in male fertilization success and our understanding of how selection shapes sperm morphology.

## Figures and Tables

**Table 1:** Summary of model selection results examining total male paternity success (*n* = 67 males) as sum of all offspring produced in a breeding season. CP volume = cloacal protuberance volume, Sperm F:H ratio = ratio of sperm flagellum length to head length, SD (Total sperm length) = standard deviation of total sperm length, Plumage color = red/back vs. brown plumage, Year = 2011 vs. 2013. Coefficients (± S.E.) are standardized and are on logit scale. Models with ΔAICc < 2 shown for reference. See Table S6 for full model output.

Model	Intercept	CP Volume (mm^3^)	Sperm F:H Ratio	Total Sperm Length (μm): Linear	SD (Total Sperm Length)	Total Sperm Length (μm): Quadratic	Helpers Present (Y/N)	Incestuous Pairing (Y/N)	Number of Neighbors	Plumage Color	Year	ΔAICc	Model Weight
1	−0.59 ± 0.41						0.58 ± 0.26	−1.88 ± 0.78	0.24 ± 0.10	1.03 ± 0.39	0.59 ± 0.22	0	0.06
2	−0.46 ± 0.42	0.13 ± 0.10					0.57 ± 0.26	−1.81 ± 0.77	0.23 ± 0.10	0.91 ± 0.40	0.52 ± 0.22	1.16	0.03
3	−0.53 ± 0.41					0.11 ± 0.10	0.59 ± 0.26	−1.84 ± 0.76	0.25 ± 0.10	0.98 ± 0.39	0.54 ± 0.22	1.42	0.03
4	−0.53 ± 0.41			0.11 ± 0.10			0.59 ± 0.26	−1.84 ± 0.77	0.25 ± 0.10	0.98 ± 0.39	0.54 ± 0.22	1.43	0.03

**Table 2:** Summary of model selection results examining male within-pair paternity success (*n* = 76 broods, from 67 males). CP volume = cloacal protuberance volume, Sperm F:H ratio = ratio of sperm flagellum length to head length, SD (Total sperm length) = standard deviation of total sperm length, Plumage color = red/back vs. brown plumage, Year = 2011 vs. 2013. Coefficients (± S.E.) are standardized and are on logit scale. Models with ΔAICc < 2 shown for reference. See Table S10 for full model output.

Model	Intercept	CP Volume (mm^3^)	Sperm F:H Ratio	Total Sperm Length (μm): Linear	SD (Total Sperm Length)	Total Sperm Length (μm): Quadratic	Helpers Present (Y/N)	Incestuous Pairing (Y/N)	Number of Neighbors	Plumage Color	Year	ΔAICc	Model Weight
1	−0.23 ± 0.39	1.17 ± 0.47	−0.68 ± 0.44									0	0.03
2	−0.26 ± 0.39	1.22 ± 0.47										0.4	0.03
3	−0.13 ± 0.39	1.15 ± 0.46	−0.80 ± 0.46					−2.55 ± 2.10				0.71	0.02
4	−0.83 ± 0.59	1.14 ± 0.46									1.03 ± 0.80	0.90	0.02
5	−0.23 ± 0.39	1.22 ± 0.48	−0.68 ± 0.44		−0.37 ± 0.38							1.31	0.02
6	−0.27 ± 0.39	1.28 ± 0.48			−0.37 ± 0.39							1.67	0.01
7	−0.60 ± 0.61	1.13 ± 0.46	−0.54 ± 0.46								0.65 ± 0.84	1.70	0.01
8	−0.12 ± 0.40	1.20 ± 0.47	−0.81 ± 0.46		−0.40 ± 0.38			−2.69 ± 2.12				1.93	0.01
9	−0.20 ± 0.40	1.21 ± 0.47						−1.65 ± 2.04				1.95	0.01

**Table 3:** Summary of model selection results examining male extra-pair paternity success (*n* = 30 males). CP volume = cloacal protuberance volume, Sperm F:H ratio = ratio of sperm flagellum length to head length, SD (Total sperm length) = standard deviation of total sperm length, Year = 2011 vs. 2013. Coefficients (± S.E.) are standardized and are on logit scale. Models with ΔAICc < 1 shown for reference. See Table S13 for full model output.

Model	Intercept	CP Volume (mm^3^)	Sperm F:H Ratio	Total Sperm Length (μm): Linear	SD (Total Sperm Length)	Total Sperm Length (μm): Quadratic	Helpers Present (Y/N)	Number of Neighbors	Year	ΔAICc	Model Weight
1	1.13 ± 0.35								−0.96 ± 0.42	0	0.04
2	1.41 ± 0.41						−0.65 ± 0.45		−1.09 ± 0.44	0.36	0.04
3	1.25 ± 0.36	0.31 ± 0.23							−1.25 ± 0.36	0.67	0.03
4	1.56 ± 0.42	0.36 ± 0.24					−0.73 ± 0.46		−1.37 ± 0.48	0.74	0.03
5	1.09 ± 0.35		0.33 ± 0.26						−0.80 ± 0.44	0.82	0.03
6	1.16 ± 0.35				0.26 ± 0.21				−1.06 ± 0.43	0.99	0.03

## Data Availability

All data are publicly available on dryad (details to be added upon publication) and provided in the supplementary documents.

## Supplementary Materials

The following supporting information can be downloaded at: https://www.mdpi.com/article/10.3390/cells11020231/s1, Figure S1: Pairwise relatedness (*r*) for all known first-order relatives (i.e., mother-offspring combinations, predicted pairwise *r* = 0.5) in our study population, FigureS2: Pairwise relatedness (*r*) for all social pairs in the study population across the entire study period (2010-2013), Table S1: Characteristics of microsatellite loci used for paternity analysis, Table S2: Results of the global model relating total male reproductive success (i.e., sum of all offspring produced) to sperm traits, male plumage colouration, and socio-ecological factors that may shape male mating opportunities, Table S3: Results of the global model relating male within-pair paternity success to sperm traits, male plumage colouration, and socio-ecological factors that may shape male mating opportunities, Table S4: Results of the global model relating male extra-pair paternity success to sperm traits, male plumage colouration, and socio-ecological factors that may shape male mating opportunities, Table S5: Sperm morphology in the red-backed fairy-wren, Table S15: Total sperm length in 12 species of Australian Maluridae, Datasets: sperm morphology data; total paternity dataset (i.e., sum of within-pair and extra-pair paternity), total paternity dataset (i.e., sum of all offspring sired across all broods vs. sum of offspring not sired by the focal male across all broods); within-pair paternity dataset; extra-pair paternity dataset.
