# Supplementary material for: Sperm Numbers as a Paternity Guard in a Wild Bird
Source: Cells. 2022 Jan 11;11(2):231. doi: 10.3390/cells11020231 (PMC8773506; doi:10.3390/cells11020231)
Supplement: Supplementary file 1 [file cells-11-00231-s001.zip › Rowe et al RBFW paternity SUPP_v2.pdf]

Supplementary materials

for

Sperm numbers as a paternity guard in a wild bird

Melissah Rowe, Annabel van Oort, Lyanne Brouwer, Jan T. Lifjeld, Michael S. Webster, Joseph F. Welklin, and Daniel T. Baldassarre

Detailed methods for genetic paternity analysis

We extracted DNA from blood samples using the Omega Bio-Tek EZ-96 Total DNA/RNA Isolation Kit®. All individuals at seven highly polymorphic microsatellite loci developed from different bird species (Table S1). For PCR reactions, we added 1 µl of extracted DNA to a mixture including 1.3 µl of 25 mM $MgCl_2$, 1 µl of 10x PCR buffer (Sigma), 0.2 µl of 10 mM dNTPs, 0.12 µl of each 10 mM PCR primer (one of each pair labeled with a fluorescent dye), 0.1 µl of 2.5U/µl *Taq* polymerase (Sigma Jumpstart®), and DNA water up to a total volume of 10 µl per well. We used the following PCR conditions: following an initial 3 min denaturation at 94°C, the reaction mix went through 35 cycles of 94°C for 30 sec, X°C for 30 sec, and 72°C for 1 min, ending with a cycle at 72°C for 5 min where X was the optimized annealing temperature or a touchdown procedure from 58-56°C (Table S1). After PCR, we created two mixtures for genotyping, consisting of 1 µl PCR products from either three or four loci, each labeled with a different dye (6FAM, PET, VIC, or NED), combined with 11.9 µl formamide and 0.1 µl size standard (GeneScan-500 LIZ®) for each well. PCR products were separated on an ABI Prism 3730® automated sequencer, and alleles were scored using the program GeneMapper (Applied Biosystems) and verified by eye.

To assign the paternity of each offspring, we assumed the breeding female observed at the nest was the genetic mother of all offspring in that nest, as has been confirmed in previous studies of this species (Webster et al. 2008; Baldassarre and Webster 2013). We were able to further validate this assumption by examining allele mismatches between the mother and offspring in her nest. If a given offspring were the result of brood parasitism, we would expect it to mismatch with the social mother at multiple loci. We never observed greater than two mismatches between offspring and assumed mothers and attributed these to null alleles or scoring errors. We assigned paternity using the program CERVUS 3.0 (Kalinowski et al. 2007), which determines which male in the population has the highest likelihood of siring a given offspring. CERVUS calculates a log likelihood score (LOD) for each male accounting for offspring genotype, maternal genotype, and genotype scoring errors (e.g., from null alleles). For each assignment, we used a "total evidence" (Prodöhl et al. 1998) approach to check the CERVUS assignment. In most cases, we accepted the CERVUS assignment if the male chosen had 0 or 1 mismatch with the nestling, but we rejected the CERVUS assignment and assigned paternity to a male with a lower LOD score under three circumstances: 1) if both males had similar LOD scores but the lower ranked male had fewer mismatches, 2) if both males had a single mismatch but the lower ranked male's mismatch could be explained by a null allele, and 3) if the males had the same low number of mismatches and similar LOD scores, but independent evidence indicated the lower ranked male was a more likely sire (after (Webster et al. 2004)). Independent evidence that we

considered included whether a candidate male was the social father, sired other offspring in the nest, exhibited a mismatch consistent with a scoring error, or was highly unlikely to have copulated with the female based on an unreasonably large distance between territories. Using this additional evidence likely improved the reliability of several assignments but was unlikely to affect our results because we accepted the CERVUS-assigned male in most cases (see below).

When combined, the microsatellite loci were highly polymorphic and informative for paternity analysis (mean number of alleles per locus = 11.7, mean expected heterozygosity = 0.69, Table 1). Allele frequencies did not deviate from Hardy-Weinberg expectations, but two loci (*Mcy2* and *Smm7*) had an estimated null allele frequency greater than 0.05, which was accounted for in subsequent paternity assignments. The average probability of excluding a randomly chosen male as the sire was high, with a combined exclusion probability of 0.998.

Table S1: Characteristics of microsatellite loci used for paternity analysis. Probability of exclusion is the probability of excluding a randomly selected, unrelated male as the sire given the genotype of the offspring and mother. References for primers are as follows: all *Mcy* loci from (Double et al. 1997), *Msp6* from (Webster et al. 2004), *Ase9* from (Richardson et al. 2000), and *Smm7* from (Maguire et al. 2006).

Locus	Annealing temperature (°C)	# Alleles	Expected heterozygosity (*He*)	Observed heterozygosity (*Ho*)	Prob. of exclusion	Null allele frequency
Mcy1	55-58	10	0.717	0.737	0.509	-0.021
Mcy2	61	5	0.088	0.076	0.044	0.071
Mcy4	55-58	12	0.822	0.761	0.66	0.035
Mcy7	61	16	0.806	0.820	0.647	0.014
Msp6	55-58	8	0.698	0.686	0.463	0.001
Ase9	61	11	0.788	0.766	0.62	0.015
Smm7	61	20	0.810	0.810	0.802	0.053

Pairwise relatedness

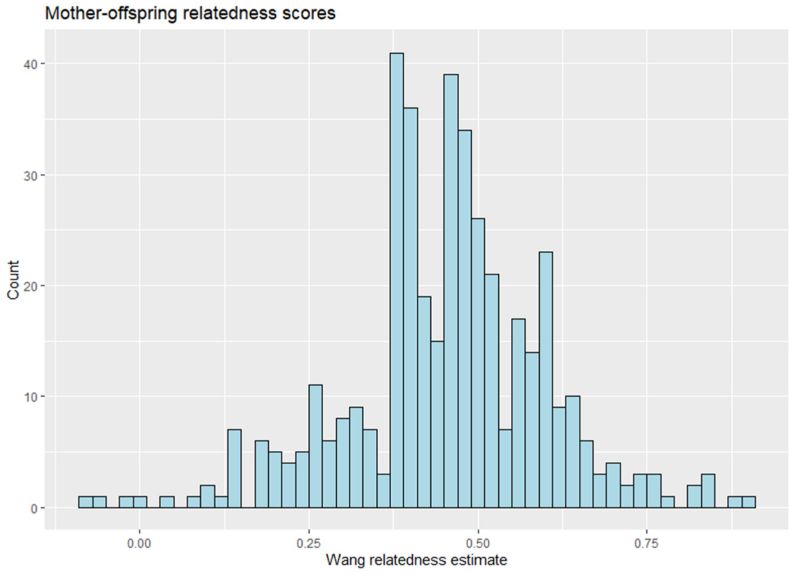

Figure S1. Pairwise relatedness (*r*) for all known first-order relatives (i.e., mother-offspring combinations, predicted pairwise *r* = 0.5) in our study population. Relatedness of known first-order relatives was r = 0.45 ± 0.15 (mean ± sd; range -0.08 to 0.91, n = 420 mother-offspring combinations).

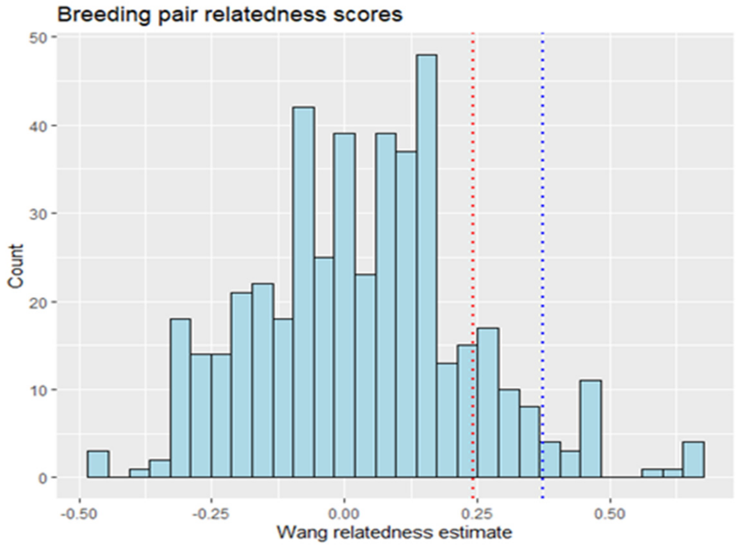

Figure S2. Pairwise relatedness (*r*) for all social pairs in the study population across the entire study period (2010-2013). Relatedness of social pairs was generally low (mean ± sd = 0.0296 ± 0.2, n = 221 pairs). Red dashed line indicates cut-off value or $r \geq 0.243$. Blue dashed line indicates cut-off value of $r \geq 0.375$ (see main text for details of cut-off values).

<u>Result of the global models examining the relationship between reproductive success and sperm traits</u>

Total Paternity Success

Total paternity success was significantly associated with male plumage colour, the number of neighbours, whether or not helpers were present, whether or not a male was paired incestuously, and year (Table S2). Specifically, males had greater total reproductive success when they bred in red/black nuptial plumage and when helpers were present in the social group. Males also gained greater reproductive success when the number of neighbouring territories was higher, whereas male reproductive success was lower when the male was paired incestuously with the social female. Total reproductive success also differed between the study years.

Within-pair paternity

In our global model, we found a positive association between sperm numbers (cloacal protuberance volume) and within-pair paternity success in male red-backed fairy-wrens (Table S3).

Extra-pair paternity

In our global model, we found a significant effect of year on extra-pair paternity success in male red-backed fairy-wrens (Table S4). Specifically, extra-pair paternity rates were lower in 2013 than 2011.

Table S2. Results of the global model relating total male reproductive success (i.e., sum of all offspring produced) to sperm traits, male plumage colouration, and socio-ecological factors that may shape male mating opportunities. Significant variables in bold.

**Fixed effects**	**Estimate (SE)**	***z***	***p***
Intercept	-0.40 (0.43)	-0.94	0.35
Total sperm length	-5.09 (8.92)	-0.57	0.57
Total sperm length (quadratic term)	5.18 (8.91)	0.58	0.56
Standard deviation (sperm length)	0.02 (0.10)	0.22	0.83
Flagellum:head ratio	0.04 (0.11)	0.36	0.72
Cloacal protuberance volume	0.12 (0.10)	1.16	0.25
**Male plumage colour** (red/black)	**0.84 (0.41)**	**2.05**	**0.041**
**Year** (2013)	**0.51 (0.24)**	**2.14**	**0.033**
**Incestuously paired** (Yes)	**-1.78 (0.78)**	**-2.30**	**0.021**
**Helpers present** (Yes)	**0.58 (0.26)**	**2.28**	**0.023**
**Number of neighbours**	**0.25 (0.10)**	**0.10**	**0.016**

Table S3. Results of the global model relating male within-pair paternity success to sperm traits, male plumage colouration, and socio-ecological factors that may shape male mating opportunities. Significant variables in bold.

**Fixed effects**	**Estimate (SE)**	***z***	***p***
Intercept	-1.20 (1.27)	-0.95	0.34
Total sperm length	-11.22 (35.19)	-0.32	0.75
Total sperm length (quadratic term)	11.22 (35.21)	0.32	0.-75
Standard deviation (sperm length)	-0.38 (0.39)	-0.96	0.34
Flagellum:head ratio	-0.68 (0.48)	-1.42	0.16
**Cloacal protuberance volume**	**1.06 (0.48)**	**2.21**	**0.027**
Male plumage colour (red/black)	0.46 (1.27)	0.36	0.72
Year (2013)	0.89 (0.90)	0.99	0.32
Incestuously paired (Yes)	-3.24 (2.19)	-1.48	0.14
Helpers present (Yes)	1.04 (1.05)	0.99	0.32
Number of neighbours	-0.13 (0.41)	-0.32	0.75
	**Variance**	**SD**	**n**
Pair ID	5.193	2.28	76

Table S4. Results of the global model relating male extra-pair paternity success to sperm traits, male plumage colouration, and socio-ecological factors that may shape male mating opportunities. Significant variables in bold.

**Fixed effects**	**Estimate (SE)**	***z***	***p***
Intercept	1.71 (0.47)	3.60	0.001
Total sperm length	13.34 (18.45)	0.72	0.47
Total sperm length (quadratic term)	-13.12 (18.40)	-0.71	0.48
Standard deviation (sperm length)	0.29 (0.24)	1.22	0.22
Flagellum:head ratio	0.44 (0.28)	1.58	0.11
Cloacal protuberance volume	0.32 (0.26)	1.24	0.21
**Year (2013)**	**-1.58 (0.57)**	**-2.75**	**0.006**
Helpers present (Yes)	-0.53 (0.54)	-0.99	0.32
Number of neighbours	-0.13 (0.27)	-0.47	0.64

Sperm morphology in the red-backed fairy-wren

Table S5. Sperm morphology in the red-backed fairy-wren. Total sperm length, length of the different sperm components, and the within-male coefficient of variation (CVwm) in sperm length across all male red-backed fairy-wrens, as well as total sperm length and length of the different sperm components for the different phenotypic classes of males (red/black breeders, brown breeders, and helpers). F:H is the ratio of sperm flagellum length to head length.

	Total sperm length (μm)	Head length (μm)	Midpiece length (μm)	Flagellum length (μm)	F:H ratio	n
All males	89.34 ± 1.94	17.06 ± 0.78	14.98 ± 0.91	72.28 ± 1.69	4.25 ± 0.21	130
All males: CVwm	2.08	4.76	6.22	2.33		130
Red/black breeders	89.44 ± 2.03	17.11 ± 0.77	15.04 ± 0.94	72.33 ± 1.79	4.24 ± 0.21	99
Brown breeders	89.36 ± 1.52	17.04 ± 0.81	14.82 ± 0.78	72.33 ± 1.37	4.27 ± 0.23	20
Helpers	87.64 ± 1.94	16.02 ± 0.41	14.49 ± 1.16	71.63 ± 1.75	4.48 ± 0.13	5

Sperm morphology data for 12 species in the Australian Maluridae

Table S15. Total sperm length in 12 species of Australian Maluridae. Measurements based on data from 10 cells per male. Shown are mean ± standard deviation.

Species	Total sperm length (µm)	Number of males
Southern emu-wren (*Stipiturus malachurus*)	75.43 ± 3.00	7
Striated grasswren (*Amytornis striatus*)	82.12 ± 2.16	8
Purple-crowned fairy-wren (*M. coronatus*)	85.28 ± 2.23	5
Splendid fairy-wren (*M. splendens*)	85.53 ± 1.47	23
Superb fairy-wren (*M. cyaneus*)	86.76 ± 2.13	15
Red-backed fairy-wren (*M. melanocephalus*)	89.34 ± 1.94	130
White-winged fairy-wren (*M. leucopterus*)	87.87 ± 1.69	11
Variegated fairy-wren (*M. lamberti*)	86.70 ± 1.76	25
Purple-backed fairy-wren (*M. assimilis*)	86.99 ± 1.14	9
Blue-breasted fairy-wren (*M. pulcherrimus*)	88.38 ± 2.07	16
Lovely fairy-wren (*M. amabilis*)	90.04 ± 1.55	6
Red-winged fairy-wren (*M. elegans*)	90.63 ± 1.97	15

R code for analyses of paternity

```
## Required libraries
library(lme4)
library(car)
library(MuMIn)
library(DHARMa)
library(MASS)

#Dataframes
TP <- read.csv("DataframeTPCount.csv") #total paternity success as sum WPP and EPY
TPPoff <- read.csv("DataframeTPProportion.csv") #total paternity success as proportion of offspring gained
WPP <- read.csv("DataframeWPP.csv") #within-pair paternity success at clutch level
EPP <- read.csv("DataframeEPP.csv") #extra-pair paternity success


## TOTAL PATERNITY ##

#### Relationship between total paternity (sum of WPP and EPP) and sperm traits + covariates

globalTPINC.nbin2011.375 <- glm.nb(TP ~ scale(TSL) + scale(TSL^2) + scale(TSL_sd)+ scale(F_to_H_ratio) + scale(CP_Vol) + factor(Plumage) + factor(Year) + factor(Inc.375) + factor(helpers) + scale(Number_of_Neighbours), data=TP)
summary(globalTPINC.nbin2011.375)
vif(globalTPINC.nbin2011.375)
options(na.action = "na.fail")

## test to see if interaction between plumage colour and CP volume should be included in global model
globalTPINC.nbin2011.375_B <- glm.nb(TP ~ scale(TSL) + scale(TSL^2) + scale(TSL_sd)+ scale(F_to_H_ratio) + scale(CP_Vol) + factor(Plumage) + factor(Year) + factor(Inc.375) + factor(helpers) + scale(Number_of_Neighbours) + factor(Plumage)*scale(CP_Vol), data=TP)
summary(globalTPINC.nbin2011.375_B)
anova(globalTPINC.nbin2011.375,globalTPINC.nbin2011.375_B) # interactions doesn't improve the model, don't include.
AICc(globalTPINC.nbin2011.375,globalTPINC.nbin2011.375_B)

##model assumptions testing for global model
(DispTP11 <- testDispersion(globalTPINC.nbin2011.375))
simTP11 <- simulateResiduals(fittedModel = globalTPINC.nbin2011.375, plot = T)

##model selection approach: total paternity
DredgeTP2011.375<- dredge(globalTPINC.nbin2011.375, rank="AICc")
head(DredgeTP2011.375, 15)
modelsDredgeTPNEW2011.375 <- get.models(DredgeTP2011.375, subset = delta < 4)
```

```
summary(modelsDredgeTPNEW2011.375[[1]])

write.csv(DredgeTP2011.375, file="TPcount.csv")

##model assumptions testing for best model
(DispTP11 <- testDispersion(modelsDredgeTPNEW2011.375[[1]]))
simTP11 <- simulateResiduals(fittedModel = modelsDredgeTPNEW2011.375[[1]], plot = T)

## try model with top value of CP removed
newTP<-subset(TP, CP_Vol < 200)

#model with new dataframe (minus 1 extreme CP value)
globalTP_newTP <- glm.nb(TP ~ scale(TSL) + scale(TSL^2) + scale(TSL_sd)+
scale(F_to_H_ratio) + scale(CP_Vol) + factor(Plumage) + factor(Year) + factor(Inc.375) +
factor(helpers) + scale(Number_of_Neighbours), data=newTP)
summary(globalTP_newTP)
vif(globalTP_newTP)
options(na.action = "na.fail")

##model assumptions testing for global model
(DispTP11 <- testDispersion(globalTP_newTP))
simTP11 <- simulateResiduals(fittedModel = globalTP_newTP, plot = T)

##model selection approach: total paternity
DredgeTP_newTP<- dredge(globalTP_newTP, rank="AICc")
head(DredgeTP_newTP, 15)
modelsDredgeTP_newTP <- get.models(DredgeTP_newTP, subset = delta < 4)
summary(modelsDredgeTP_newTP[[1]])

write.csv(DredgeTP_newTP, file="TPcount_modCP.csv")


##Repeat main anlaysis with alternate r cut-off r 0.243
globalTPINC.nbin2011.243 <- glm.nb(TP ~ scale(TSL) + scale(TSL^2) + scale(TSL_sd)+
scale(F_to_H_ratio) + scale(CP_Vol) + factor(Plumage) + factor(Year) + factor(Inc.243) +
factor(helpers) + scale(Number_of_Neighbours), data=TP)
summary(globalTPINC.nbin2011.243)
vif(globalTPINC.nbin2011.243)
options(na.action = "na.fail")

##model selection approach: total paternity r 0.243
DredgeTP2011.243<- dredge(globalTPINC.nbin2011.243, rank="AICc")
head(DredgeTP2011.243, 15)
modelsDredgeTPNEW2011.243 <- get.models(DredgeTP2011.243, subset = delta < 4)
summary(modelsDredgeTPNEW2011.243[[1]])

write.csv(DredgeTP2011.243, file="TPcount_243.csv")
```

```
#### Relationship between total paternity (proportion response) and sperm traits +
covariates

cbTPGloPost <- glm(cbind(TP,(Chicks_Year-TP)) ~ scale(TSL) + scale(TSL^2) + scale(TSL_sd)
+ scale(F_to_H_ratio) + scale(CP_Vol) + factor(Year) + factor(Plumage) + factor(Inc.375) +
factor(helpers) + scale(Number_of_Neighbours), data=TPPoff, family=binomial(link="logit"))
summary(cbTPGloPost)
vif(cbTPGloPost)
options(na.action = "na.fail")

#test assumptions of global model
(Dispersion_TP_global <- testDispersion(cbTPGloPost ))
simulationOutput_globalTP  <- simulateResiduals(fittedModel = cbTPGloPost, plot = T)

# test to see if interaction between plumage colour and CP volume should be included in
global model
cbTPGloPost_A <- glm(cbind(TP,(Chicks_Year-TP)) ~ scale(TSL) + scale(TSL^2) +
scale(TSL_sd) + scale(F_to_H_ratio) + scale(CP_Vol) + factor(Year) + factor(Plumage) +
factor(Inc.375) + factor(helpers) + scale(Number_of_Neighbours) +
factor(Plumage)*scale(CP_Vol), data=TPPoff, family=binomial(link="logit"))
summary(cbTPGloPost_A)
anova(cbTPGloPost,cbTPGloPost_A, test="Chisq") # interactions doesn't improve the model,
don't include.

#model selection approach
DrTPP <- dredge(cbTPGloPost, rank="AICc")
head(DrTPP, 15)
MoDrTPP <- get.models(DrTPP, subset = delta < 4)
summary(MoDrTPP[[1]])
r.squaredGLMM(MoDrTPP[[1]])

##export model selection outputs
write.csv(DrTPP, file="TPPoff.csv")

#test assumptions about top model
(DiTP3 <- testDispersion(MoDrTPP[[1]]))
SiTP3 <- simulateResiduals(fittedModel = MoDrTPP[[2]], plot = T)


## WITHIN-PAIR PATERNITY ##

#### Examining relationship between within-pair paternity and sperm traits + covariates -
global model
globalWP.375P <- glmer(cbind(WPY_Clutch, EPY_in_Clutch) ~  scale(TSL) + scale(TSL^2) +
scale(TSL_sd) + scale(F_to_H_ratio) + scale(CP_Vol) + factor(Plumage) + factor(Year) +
```

```
factor(Inc.375) + factor(helpers) + scale(Number_of_Neighbours) + (1|PairID) ,  data=WPP,
family=binomial(link="logit"), control=glmerControl(optimizer="bobyqa",
optCtrl=list(maxfun=100000)))
summary(globalWP.375P)
vif(globalWP.375P)
options(na.action = "na.fail")

#test model assumptions of the global model
(Dispersion_WPP_global <- testDispersion(globalWP.375P ))
simulationOutput_globalWP.375P  <- simulateResiduals(fittedModel = globalWP.375P, plot
= T) #model is fine

#Test to see if interaction between male plumage colour and CP vol should be included in
global model
globalWP.375P_A <- glmer(cbind(WPY_Clutch, EPY_in_Clutch) ~  scale(TSL) + scale(TSL^2)
+ scale(TSL_sd) + scale(F_to_H_ratio) + scale(CP_Vol) + factor(Plumage) + factor(Year) +
factor(Inc.375) + factor(helpers) + scale(Number_of_Neighbours) +
factor(Plumage)*scale(CP_Vol) + (1|PairID) ,  data=WPP, family=binomial(link="logit"),
control=glmerControl(optimizer="bobyqa", optCtrl=list(maxfun=100000)))
summary(globalWP.375PA)
anova(globalWP.375P,globalWP.375P_A) ## don't include the interaction, use more
parsimonious model without interaction
AICc(globalWP.375P,globalWP.375P_A)

#model selection approach
DredgeWPNEW <- dredge(globalWP.375P , rank="AICc")
head(DredgeWPNEW, 25)
modelsDredgeWPNEW <- get.models(DredgeWPNEW, subset = delta < 4)
summary(modelsDredgeWPNEW[[1]])
r.squaredGLMM(modelsDredgeWPNEW[[2]])
predict1<-predict(modelsDredgeWPNEW[[1]])

WPPSDTSL$predict<-predict1
write.csv(WPPSDTSL,file="WPPpredict.csv")

##export model selection outputs
write.csv(DredgeWPNEW, file="WPP.csv")
write.csv(predict1,file="predictWPPtopmodel.csv")

#test assumptions about top model
(Dispersion_WPP_NEW <- testDispersion(modelsDredgeWPNEW[[1]]))
simulationOutput_WPP_NEW <- simulateResiduals(fittedModel =
modelsDredgeWPNEW[[1]], plot = T)


## Repeat this analysis using 0.243 as incestuous cutoff
```

```
globalWP.243P <- glmer(cbind(WPY_Clutch, EPY_in_Clutch) ~  scale(TSL) + scale(TSL^2) +
scale(TSL_sd) + scale(F_to_H_ratio) + scale(CP_Vol) + factor(Plumage) + factor(Year) +
factor(Inc.243) + factor(helpers) + scale(Number_of_Neighbours) + (1|PairID) ,  data=WPP,
family=binomial(link="logit"), control=glmerControl(optimizer="bobyqa",
optCtrl=list(maxfun=100000)))
summary(globalWP.243P)
vif(globalWP.243P)
options(na.action = "na.fail")

##model selection approach using 0.243 incestuous pairr cutoff
DredgeWPNEW_Inc.243 <- dredge(globalWP.243P , rank="AICc")
head(DredgeWPNEW_Inc.243, 25)
modelsDredgeWPNEW_Inc.243 <- get.models(DredgeWPNEW_Inc.243, subset = delta < 4)
summary(modelsDredgeWPNEW_Inc.243[[1]])
r.squaredGLMM(modelsDredgeWPNEW_Inc.243[[1]])

##export model selection outputs with Incestuous pairs cutoff as 0.243
write.csv(DredgeWPNEW_Inc.243, file="WPP_Inc.243.csv")

##test assumptions about top model with Incestuous pairs cutoff as 0.243
(Dispersion_WPP_NEW <- testDispersion(modelsDredgeWPNEW_Inc.243[[1]]))
simulationOutput_WPP_NEW <- simulateResiduals(fittedModel =
modelsDredgeWPNEW_Inc.243[[1]], plot = T)


## try model with top value of CP removed
newWPP<-subset(WPP, CP_Vol < 200)

globalWP_NEWWPP <- glmer(cbind(WPY_Clutch, EPY_in_Clutch) ~  scale(TSL) +
scale(TSL^2) + scale(TSL_sd) + scale(F_to_H_ratio) + scale(CP_Vol) + factor(Plumage) +
factor(Year) + factor(Inc.375) + factor(helpers) + scale(Number_of_Neighbours) + (1|PairID) ,
data=newWPP, family=binomial(link="logit"), control=glmerControl(optimizer="bobyqa",
optCtrl=list(maxfun=100000)))
summary(globalWP_NEWWPP)
vif(globalWP_NEWWPP)
options(na.action = "na.fail")

#test model assumptions of the new global model
(Dispersion_WPP_global <- testDispersion(globalWP_NEWWPP))
simulationOutput_globalWP_NEWWPP  <- simulateResiduals(fittedModel =
globalWP_NEWWPP, plot = T) #model is fine

#model selection approach
DredgeWPNEW_NEWWPP <- dredge(globalWP_NEWWPP , rank="AICc")
head(DredgeWPNEW_NEWWPP, 25)
modelsDredgeWPNEW_NEWWPP <- get.models(DredgeWPNEW_NEWWPP, subset =
delta < 4)
```

```
summary(modelsDredgeWPNEW_NEWWPP[[1]])

write.csv(DredgeWPNEW_NEWWPP, file="WPP_reducedCP.csv")

## EXTRA-PAIR PATERNITY ##

#### Relationship between extra-pair paternity and sperm traits + covariates
GLMEPPBrightNEW1 <- glm(cbind(sumEPY, NoEPY) ~  scale(TSL) + scale(TSL^2) +
scale(TSL_sd) + scale(F_to_H_ratio) + scale(CP_Vol) + factor(Year) + factor(helpers) +
scale(Number_of_Neighbours),  data=EPP, family=binomial(link="logit"))
summary(GLMEPPBrightNEW1)
vif(GLMEPPBrightNEW1)
options(na.action = "na.fail")

#test model assumptions of the global model
(Dispersion_EPP_global <- testDispersion(GLMEPPBrightNEW1))
simulationOutput_globalEPP  <- simulateResiduals(fittedModel = GLMEPPBrightNEW1,
plot = T) #model is fine

#model selection approach
EPPDredgeBrightNew1 <- dredge(GLMEPPBrightNEW1, rank="AICc")
head(EPPDredgeBrightNew1, 25)
modelsEPPDredgeBrightNew1 <- get.models(EPPDredgeBrightNew1, subset = delta < 4)
summary(modelsEPPDredgeBrightNew1[[1]])
r.squaredGLMM(modelsEPPDredgeBrightNew1[[1]])

##export model selection outputs
write.csv(EPPDredgeBrightNew1, file="EPP.csv")

## test model assumptions
(Dispersion_EPP_BrightNew1 <- testDispersion(modelsEPPDredgeBrightNew1[[1]]))
simulationOutputEPPBrightNew1 <- simulateResiduals(fittedModel =
modelsEPPDredgeBrightNew1[[1]], plot = T)


##Repeat EPP with incestuous pair cut-off as r > 0.243
GLMEPPBrightNEW1.243 <- glm(cbind(sumEPY, NoEPY) ~  scale(TSL) + scale(TSL^2) +
scale(TSL_sd) + scale(F_to_H_ratio) + scale(CP_Vol) + factor(Year) + factor(Inc.243) +
factor(helpers) + scale(Number_of_Neighbours),  data=EPP, family=binomial(link="logit"))
summary(GLMEPPBrightNEW1.243)
vif(GLMEPPBrightNEW1.243)
options(na.action = "na.fail")

##model selection approach using 0.243 incestuous pair cutoff

DredgeGLMEPPBrightNEW1.243 <- dredge(GLMEPPBrightNEW1.243, rank="AICc")
head(DredgeGLMEPPBrightNEW1.243, 25)
```

```
modelsDredgeGLMEPPBrightNEW1.243 <- get.models(DredgeGLMEPPBrightNEW1.243, subset = delta < 4)
summary(modelsDredgeGLMEPPBrightNEW1.243[[8]])
r.squaredGLMM(modelsDredgeGLMEPPBrightNEW1.243[[1]])

##export model selection outputs
write.csv(DredgeGLMEPPBrightNEW1.243, file="EPP_In234.csv")

## test model assumptions
(Dispersion_DredgeGLMEPPBrightNEW1.243 <- testDispersion(modelsDredgeGLMEPPBrightNEW1.243[[1]]))
simulationOutputEPPBrightNew1.243 <- simulateResiduals(fittedModel = modelsDredgeGLMEPPBrightNEW1.243[[1]], plot = T)
```
